# Draft Genome Sequences of Flavobacterium columnare Strains ARS1 and BGFS27, Isolated from Channel Catfish (*Ictalurus punctatus*)

**DOI:** 10.1128/MRA.00648-19

**Published:** 2019-06-27

**Authors:** Wenlong Cai, C. R. Arias

**Affiliations:** a School of Fisheries, Aquaculture, and Aquatic Sciences, Auburn University, Auburn, Alabama, USA; b HOPLITE Research Group, Department of Pathology and Microbiology, Atlantic Veterinary College, University of Prince Edward Island, Charlottetown, PE, Canada; University of Rochester School of Medicine and Dentistry

## Abstract

Flavobacterium columnare strain BGFS27 was isolated from an apparently healthy wild channel catfish (Ictalurus punctatus) collected from the Mobile River in 2005. *F. columnare* strain ARS1 was isolated from a channel catfish suffering from columnaris disease in a commercial farm in 1996. BGFS27 belongs to genomovar II (genetic group 2), while ARS1 belongs to genomovar III (genetic group 3). Here, we report the draft genome sequences of *F. columnare* BGFS27 and ARS1, obtained by PacBio sequencing.

## ANNOUNCEMENT

Flavobacterium columnare is a Gram-negative bacterium that belongs to the *Flavobacteriaceae* family within the phylum *Bacteroidetes*. *F. columnare* is the causative agent of columnaris disease (1, 2), which can affect a broad range of fish hosts, including wild, cultured, and ornamental species (3). *F. columnare* is genetically heterogeneous and has been divided into 6 genomovars based on 16S rRNA gene sequencing and DNA-DNA hybridization (4–7). Based on empirical studies, strains of genomovar II have demonstrated higher virulence toward channel catfish. In addition, based on epidemiological surveys, genomovar II strains are responsible for the majority of the columnaris outbreaks affecting channel catfish and sport fish species in Alabama (8). Recently, LaFrentz et al. proposed the use of four genetic groups to facilitate typing based on 16S rRNA gene sequences and clarify the epidemiology of columnaris disease (9). BGFS27 belongs to genomovar II (genetic group 2). It is a highly virulent strain for channel catfish that our group used as the parent strain to develop a modified live vaccine (10). ARS1 was initially ascribed to genomovar I and has been used as a low-virulence strain for catfish by our group and others (11–14). However, after the 16S rRNA gene restriction fragment length polymorphism (RFLP) typing method was standardized, the strain was ascribed to genomovar III (genetic group 3) (9).

Bacteria were cultured at 28°C in modified Shield medium for 48 h with shaking at 200 rpm (15). The total genomic DNA was isolated using a Qiagen DNeasy blood and tissue kit (Qiagen, MD, USA) following the manufacturer’s instructions, including the RNase incubation step. All the DNA samples were quantified using a NanoDrop ND-1000 spectrophotometer (Thermo Fisher Scientific, Wilmington, DE, USA) and run on 1% agarose gel for the integrity check. The library preparation and sequencing were conducted at the University of Washington PacBio Sequencing Service Center (Seattle, WA). DNA was sheared, and the libraries were prepared using the standard 20-kb library protocol of the SMRTbell template prep kit 1.0 (PacBio, Menlo Park, CA) without size select. The libraries were sequenced using the PacBio long-read sequencing RS II platform with P6-C4 chemistry. A total of 434,427 subreads with an *N*_50_ value of 4,264 bp were obtained from strain BGFS27, and 418,658 subreads with an *N*_50_ value of 3,364 bp were obtained for ARS1. Genome assembly of the filtered reads was performed using the PacBio Hierarchical Genome Assembly Process (HGAP) 2.3 pipeline with default settings using the *de novo* assembly protocol, and the reads were polished using Quiver. The *F. columnare* BGFS27 and ARS1 draft genome sequences comprised 10 and 4 contigs with 183× and 182× coverage, respectively. The *N*_50_ values for the BGFS27 and ARS1 assemblies were 1.65 Mb and 2.22 Mb, respectively. Four contigs with a total size of 148 kb in the BGFS27 draft genome sequence were manually removed due to a low-quality value of less than QV25, resulting in 6 contigs with a mean quality value of QV48.5 that were used as the final draft genome sequence. The BGFS27 draft genome size was 3.53 Mbp with a GC content of 31.1%. The ARS1 draft genome comprised 4 contigs with a mean quality value of QV48.8. The ARS1 draft genome size was 3.36 Mbp with a GC content of 31.0%.

The contigs were annotated with Rapid Annotations using Subsystems Technology (RAST) for bacteria (16). BGFS27 contained 3,270 coding sequences, 242 subsystems, and 116 RNAs, while ARS1 contained 2,903 coding sequences, 235 subsystems, and 104 RNAs. The average nucleotide identity (ANI) was 85.69% per EDGAR calculations (17), considerably below the typical cutoff point of 95% for species delineation (18).

### Data availability.

The draft genome sequences were deposited at DDBJ/ENA/GenBank under the accession numbers RQSM00000000 and RWGX00000000. The versions described in this paper are the first versions. The raw sequencing reads have been deposited in the Sequence Read Archive under the accession numbers SRX5190172 and SRX5189924.
